# Nitrate Intake Does Not Influence Bladder Cancer Risk: The Netherlands Cohort Study

**DOI:** 10.1289/ehp.9098

**Published:** 2006-07-13

**Authors:** Maurice P. Zeegers, Roel F.M. Selen, Jos C.S. Kleinjans, R. Alexandra Goldbohm, Piet A. van den Brandt

**Affiliations:** 1 Department of Public Health and Epidemiology, University of Birmingham, Birmingham, United Kingdom; 2 Comprehensive Cancer Institute Limburg, Department of General Practice, Catholic University of Leuven, Leuven, Belgium; 3 Department of Epidemiology and; 4 Department of Health Risk Analysis and Toxicology, Maastricht University, Maastricht, the Netherlands; 5 Department of Food and Chemical Risk Analysis, TNO Quality of Life, Zeist, the Netherlands

**Keywords:** bladder cancer, cohort study, epidemiology, etiology, nitrate

## Abstract

**Objectives:**

*N*-nitroso compounds, endogenously formed from nitrate-derived nitrite, are suspected to be important bladder carcinogens. However, the association between nitrate exposure from food or drinking water and bladder cancer has not been substantially investigated in epidemiologic studies.

**Methods:**

We evaluated the associations between nitrate exposure and bladder cancer in the Netherlands Cohort Study, conducted among 120,852 men and women, 55–69 years of age at entry. Information on nitrate from diet was collected via a food frequency questionnaire in 1986 and a database on nitrate content of foods. Individual nitrate exposures from beverages prepared with tap water were calculated by linking the postal code of individual residence at baseline to water company data. After 9.3 years of follow-up and after excluding subjects with incomplete or inconsistent dietary data, 889 cases and 4,441 subcohort members were available for multivariate analyses. We calculated incidence rate ratios (RR) and corresponding 95% confidence intervals (CIs) using Cox regression analyses. We also evaluated possible effect modification of dietary intake of vitamins C and E (low/high) and cigarette smoking (never/ever).

**Results:**

The multivariate RRs for nitrate exposure from food, drinking water, and estimated total nitrate exposure were 1.06 (95% CI, 0.81–1.31), 1.06 (95% CI, 0.82–1.37), and 1.09 (95% CI, 0.84–1.42), respectively, comparing the highest to the lowest quintiles of intake. Dietary intake of vitamins C and E (low/high) and cigarette smoking (never/ever) had no significant impact on these results.

**Conclusion:**

Although the association between nitrate exposure and bladder cancer risk is biologically plausible, our results in this study do not support an association between nitrate exposure and bladder cancer risk.

Nitrate is a natural compound of green vegetables, such as lettuce and spinach, and root vegetables, such as beets. Nitrate is also present in drinking water (Gangolli et al. 1994; McKnight et al. 1999; van Loon et al. 1997, 1998). In the late 1990s, the concentration of nitrate in the Netherlands increased in vegetables and drinking water due to cultivation and the use of artificial fertilizers (van Loon et al. 1998), and these nitrate concentrations have remained stable to date (Versteegh et al. 2004). This continued high concentration causes growing concern because of the potential health risks of the metabolites of nitrate and because of their potential relationship with cancer.

There is still a relative deficit of epidemiologic data addressing the association between nitrate exposure and cancer risk. Most of the epidemiologic studies that are available have focused on gastric cancer risk (Boeing et al. 1991; Buiatti et al. 1990; Cantor 1997; Forman 1989; Hansson et al. 1994; Risch et al. 1985; van Loon et al. 1997), but showing little support for the supposed relationship between nitrate and gastric cancer risk. However, an association between nitrate exposure and bladder cancer risk is biologically plausible.

After ingestion, approximately 20% of nitrate is endogenously transformed to nitrite by the bacterial flora of the oral cavity (Weyer et al. 2001). Nitrite can react in the stomach with foodborne secondary amines or amides to form *N*-nitroso compounds (NOCs), depending on the availability of nitrate in the stomach (Mirvish et al. 1987; van Loon et al. 1998; Walker 1990; Weyer et al. 2001). Because approximately 70% of the orally ingested nitrate is excreted in the urine, nitrosation may also occur in the bladder (Gulis et al. 2002; Preston-Martin and Correa 1989; Weyer et al. 2001). Several lifestyle-related factors can influence nitrosation. Food components, such as vitamins C and E, may inhibit the conversion of nitrate into nitrite or block nitrosation (Council of Europe 1995), whereas other factors, such as smoking cigarettes, can promote nitrosation (Ward et al. 2003; Weyer et al. 2001). Smokers appear to have a lower concentration of nitrate in the blood because of the higher thiocyanate concentration, which can be up to four times higher than in nonsmokers (Council of Europe 1995; Mirvish et al. 1987). It is not nitrate per se but its metabolites that are potent rodent carcinogens (Tricker and Preussmann 1991), inducing several types of cancer including cancer of the stomach, colon, and lymphatic and hematopoietic system, and possibly bladder (Bogovski and Bogovski 1981; Gulis et al. 2002; Mirvish et al. 1987).

Previous epidemiologic studies focused on nitrate exposure and bladder cancer risk are sparse; also, they have addressed nitrate intake only from drinking water and have used ecologic (Gulis et al. 2002; Morales-Suarez-Varela et al. 1995) or case–control (Ward et al. 2003) designs. Only one prospective study on drinking-water nitrate intake and bladder cancer risk has been published so far (Weyer et al. 2001). In that study of the prospective Iowa Women’s Health Study, Weyer et al. (2001) found an increased risk of bladder cancer among older women exposed to relatively low drinking-water levels of > 2.46 mg/L/day nitrate, compared with women using < 2.46 mg/L/day [incidence rate ratios (RR), 2.83; 95% confidence interval (CI), 1.11–7.19]. Weyer et al. (2001), however, could not provide data on men (Kantoff 1992).

In the present study, the prospective design, the possibility of investigating nitrate in both food and drinking water, the large study population including both men and women, and the possibility of studying confounding and effect modification by environmental characteristics allowed us to study the association between nitrate intake and bladder cancer incidence in more detail in the Netherlands Cohort Study.

## Materials and Methods

### Study population

The design of the Netherlands Cohort Study (NLCS) has been reported in detail elsewhere (van den Brandt et al. 1990). Briefly, the NLCS was initiated in September 1986 and includes 58,279 men and 62,573 women 55–69 years of age at baseline. Ethical approval was obtained from the University Hospital Maastricht (Maastricht, the Netherlands) and TNO. This study population originated from 204 municipal registries throughout the country. All cohort members consented to participation by completing a mailed, self-administered questionnaire on risk factors for cancer.

For reasons of efficiency in data processing and analysis, we used the case–cohort approach (Prentice 1986). Cases were derived from the entire cohort (providing numerator information for calculation of cancer incidence rates), and accumulated person-years at risk in the total cohort were estimated using a random subcohort sample (providing denominator information for the rates). The subcohort was sampled directly after identification of all cohort members and has been followed up biennially for vital status information.

### Follow-up

The follow-up for incident bladder cancer in the entire cohort was established by computerized record linkage with the cancer registries in the Netherlands and the Dutch national database of pathology reports (PALGA). Completeness of follow-up of cancer has been estimated to be at least 96% (Goldbohm et al. 1994). The analysis is restricted to cancer incidence in 9.3 years of follow up, from September 1986 through December 1995. During this follow-up, 955 bladder cancer cases were registered.

### Nitrate intake

Each participant’s usual consumption of food and beverages during the year preceding the start of the study was assessed at baseline with a 150-item semiquantitative food frequency questionnaire specifically developed to measure nutrient contents such as nitrate. This questionnaire has been validated against a 9-day diet record (Goldbohm et al. 1994). Food composition values for nitrate were derived from the databank on contaminants in food from the State Institute for Quality Control of Agricultural Products (RIKILT; Wageningen, the Netherlands) (van Loon 1997 et al. 1998). Estimations were based on the mean nitrate contents between 1985 and 1989, in which distinction for some vegetables (e.g., endive, lettuce) was made between summer and winter. Furthermore, information on nitrate losses during preparation (washing, cutting, cooking) were considered. For several vegetables (endive, spinach, cabbage) and for potatoes, experimental data were available regarding nitrate losses during preparation (Driessen 1989; van de Worp 1987). For other vegetables, nitrate losses were estimated to be 40%. For water, we combined information about nitrate (NO^−^_3_/L) content in drinking water from all 364 pumping stations in the Netherlands in 1986 [Vereniging van Exploitanten van Waterleiding bedrijven in Nederland (VEWIN) 1989]. In this way, we could determine the nitrate concentration in drinking water for each home address by postal code. If more pumping stations delivered drinking water to a single residence, a weighted mean was calculated. To calculate the nitrate intake from water, we used the information about the amount of water, coffee, tea, and soup consumed, which where derived from the questionnaire.

### Statistical analysis

Subjects with incomplete or inconsistent dietary data were excluded from the analyses (Goldbohm et al. 1994), leaving 889 cases and 4,441 subcohort members available for analyses. Because of additional missing values for chemical water quality, 871 cases and 4,359 subcohort members were available for analyses on nitrate exposure from drinking water and total nitrate exposure.

Incidence rate ratios (RRs) and corresponding 95% confidence intervals (CIs) for bladder cancer were estimated in age- and sex-adjusted and multivariable case–cohort analyses using the Cox proportional hazards model (Cox 1972) processed with the Stata statistical software package (Cleves et al. 2002). We estimated SEs using the robust Huber-White sandwich estimator to account for additional variance introduced by sampling from the cohort (Lin and Wei 1989). The proportional hazards assumption was tested using the scaled Schoenfeld residuals (Schoenfeld 1982). Tests for dose–response trends in risk of bladder cancer were assessed by fitting ordinal exposure variables as continuous terms. All *p*-values presented are two-sided. Statistical tests for interaction were based on Wald statistics of interaction product terms.

The following variables have been considered as potential confounding factors, based on earlier analyses (Zeegers et al. 2001a, 2001b, 2001c, 2001d, 2002a, 2002b): age (years), alcohol consumption (grams per day), coffee consumption (cups per day), tea consumption (cups per day), water consumption (liters per day), vegetable consumption (grams per day), fruit consumption (grams per day), dietary vitamin C and E intake (milligrams per day), cigarette smoking (never/ever), current cigarette smoking (percentage yes), smoking amount (cigarettes per day), smoking duration (years of cigarette smoking), first degree family history of bladder cancer (percentage yes), and high-risk occupational exposure to dye, rubber, leather or vehicle fumes (percentage ever). Furthermore, other variables were tested as possible effect modifiers between nitrate exposure from diet and drinking water (low/high exposure) and bladder cancer risk: intake of dietary vitamins C and E (low/high) and smoking of cigarettes (never/ever). Noninvasive tumors [carcinoma *in situ* (Tis), noninvasive papillary carcinoma (Ta), and submucosal invasion (T1)] were evaluated separately from invasive tumors [invasion of muscle (T2)–invasion of prostate, uterus, vagina, pelvic wall, or abdominal wall (T4)] to test whether bladder cancer progression is associated with nitrate intake.

## Results

Table 1 presents the average baseline nitrate levels from food and drinking water and the estimated total nitrate intake per day among bladder cancer cases and the subcohort members. Although exposure to nitrate from food was comparable between cases and subcohort members, nitrate exposure from drinking water and estimated total nitrate intake appeared to be somewhat higher among cases. Of the potential confounders, age, alcohol intake, coffee consumption, water consumption, dietary vitamin E intake, cigarette smoking amount, and smoking duration were somewhat higher among cases than among subcohort members, whereas tea consumption, dietary vitamin C intake, and fruit and vegetable consumption were higher among subcohort members. Most differences, however, were nonsignificant. As expected, men were overrepresented among cases (86.2%), whereas the male/female distribution was about equal among subcohort members. Most of the cases (87.8%) had smoked cigarettes, compared to only 64.1% of the subcohort members. This contrast was stronger for current smoking at baseline, when 44.2% of the cases and 28.2% of the subcohort members smoked. The frequency of having first degree family members with bladder cancer or having worked in a high risk occupation were very low among both cases and subcohort members (Table 1).

Cox regression analyses showed that only age, sex, and cigarette smoking were confounders; therefore, we adjusted for these in subsequent multivariate analyses. The RRs for nitrate exposure from food, drinking water, and estimated total nitrate exposure are presented in Table 2. When adjusted for age and sex, the RR for nitrate exposure from food was 1.01 (95% CI, 0.79–1.29; *p*-trend = 0.72), comparing the highest quintile with the lowest quintile. After adjustment for cigarette smoking, the RR increased slightly to 1.06 (95% CI, 0.81–1.37; *p*-trend = 0.96). Additional adjustment for nitrate from drinking water did not change these estimates substantially. When comparing the highest quintile with the lowest quintile of nitrate exposure from drinking water, the RR was 1.11 (95% CI, 0.87–1.41; *p*-trend = 0.14), after adjusting for age and sex. When adjusted for age, sex and smoking, the RR decreased to 1.06 (95% CI, 0.82–1.37; *p*-trend = 0.23). This RR did not change further after additional adjustment for nitrate exposure from food. The age- and sex-adjusted RR for estimated total nitrate exposure was 1.03 (95% CI, 0.80–1.32; *p*-trend = 0.86), when comparing the highest quintile with the lowest quintile of exposure. When adjusted for age, sex and smoking, this RR increased to 1.06 (95% CI, 0.82–1.38; *p*-trend = 0.77). These results were similar for men and women for nitrate from food (*p* = 0.62), nitrate from water (*p* = 0.14) and total nitrate (*p* = 0.74) (data not shown).

Vitamin C (*p* = 0.63), vitamin E (*p* = 0.62), and cigarette smoking (*p* = 0.11) did not appear to be significant effect modifiers in the association between nitrate exposure from food and bladder cancer risk (Table 3). Smoking was also not an effect modifier when nitrate exposure from drinking water was evaluated (Table 4).

Table 5 presents the association between nitrate exposure and transitional cell carcinoma, with respect to tumor invasiveness. The relative risks for high versus low nitrate intake from food and/or drinking water did not differ between invasive and noninvasive tumors (Table 5).

## Discussion

In the present study, we examined the association between nitrate exposure and the risk of developing bladder cancer in a prospective cohort study. Although the association between nitrate exposure and bladder cancer risk is biologically plausible, our results do not show a statistically significant association for baseline nitrate exposure from food or drinking water or on the basis of total nitrate exposure. To our knowledge, this is the first study to investigate nitrate exposure and bladder cancer risk with respect to tumor invasiveness. In these analyses, we found no significant results.

The main source of dietary nitrate intake is vegetables. However, vegetables may contain certain vitamins that can act as inhibitors of endogenous nitrosation. As a consequence, dietary intake of nitrate may not result in a substantial formation of NOCs (Ward et al. 2003), although conflicting results have been reported on this issue (Vermeer et al. 1999). On the other hand, cigarettes also contain NOCs, which could further promote the endogenous nitrosation in the body and therefore result in a substantial formation of NOCs. We have investigated both of these aspects in this study. Although plausible, interaction analyses between dietary nitrate intake and dietary intake of vitamins C and E did not yield significant results. Although vitamin C intake decreased the risk of bladder cancer marginally in this population, high vitamin E intake did not appear to decrease the risk (data not shown). Because nitrate from water is often assumed to pose a greater cancer risk than nitrate from foods that contain antioxidants and other protective nutrients, we also evaluated the effects of nitrate-contaminated water on smokers in a separate analysis. In stratified analyses, we found that smoking cigarettes did not significantly change the association between nitrate intake from either food or water and bladder cancer risk. However, our never-smoking stratum was small because of relatively high European smoking rates. It is possible that a larger nonsmoking population would yield a different answer.

A null association for nitrate from drinking water may be explained by the rather low levels of nitrate in Dutch drinking water. We assessed the intake of nitrate in drinking water from regular drinking water supplies by combining information on nitrate contents in drinking water from all water companies in the Netherlands (VEWIN 1989) with information about the distribution for the baseline year 1986. The average level of nitrate in Dutch drinking water is 1.68 mg/L (VEWIN 1989). Approximately 5% of the pumping stations have supplied drinking water with a nitrate level between 25 and 50 mg/L (van Duyvenbooden and van Matthysen 1989). Unfortunately, no information was available about dietary and water nitrate intake during follow-up. However, in a reproducibility study, Goldbohm et al. (1995) demonstrated that the single food frequency questionnaire measurement could characterize dietary habits for a period of at least 5 years. Furthermore, data from the National Institute for Public Health and the Environment (Bilthoven, the Netherlands) showed no substantial differences in average nitrate concentrations in drinking water within the last decade (Versteegh et al. 2004). Our results are consistent with those of van Loon et al. (1997) concerning nitrate intake from food and the lack of an association between drinking water nitrate intake and cancer: van Loon et al. (1997) found no associations between nitrate intake from Dutch drinking water and gastric cancer.

Some factors may have influenced the results of the present study. A possible weakness of this study is that we were not able to control for bladder infections, a possible risk factor for bladder cancer. Also, the results could have been influenced by misclassification of exposure. Dietary nitrate intake was assessed by combining information on food intake with nitrate contents in food, and intake of food was estimated with a semiquantitative food frequency questionnaire. This method has been validated against urinary measurement: the adjusted partial correlation coefficient of intake verses urinary excretion was 0.59, indicating that a self-administered dietary questionnaire can be used to measure usual nitrate intake (van den Brandt et al. 1989). To account for the relatively large variation in nitrate levels in vegetables over seasons, we calculated mean nitrate contents in vegetables from average nitrate levels in summer and winter. The prospective nature of a cohort study, together with completeness of follow-up, as has been achieved in this study, reduced the potential for recall and selection bias to a minimum. Information bias was also largely avoided because dietary habits were reported before bladder cancer was diagnosed.

Our finding of no association with bladder cancer is consistent with some of the earlier studies conducted in Slovakia (Gulis et al. 2002) and in Iowa (USA) (Ward et al. 2003). The first ecologic study, conducted in Slovakia (Gulis et al. 2002), showed no association overall; the highest standardized incidence ratio (1.22; 95% CI, 0.90–1.64) was reported among people exposed to 0–10 mg/L nitrate from drinking water. The second ecologic study, conducted in Spain (Morales-Suarez-Varela et al. 1995), reported an association with bladder cancer among males 55–75 years of age and exposed to > 50 mg/L of nitrates in drinking water (RR = 1.4; 95% CI, 0.8–2.48). A case–control study in Iowa (Ward et al. 2003) found no association with bladder cancer and nitrate levels in water supplies. The highest quartile odds ratio (OR) for women exposed to ≥ 2.48 mg/L nitrate in drinking water was 0.8 (95% CI, 0.4–1.3). The highest quartile OR for men exposed to ≥ 3.09 mg/L nitrate in drinking water was 0.5 (95% CI, 0.4–0.8). The Iowa Women’s Health Study (Weyer et al. 2001) found an increased risk of bladder cancer among older women exposed to drinking water levels of nitrate > 2.5 mg/L; the results showed an RR of 2.83 (95% CI, 1.11–7.19).

We conclude that there is no association between average nitrate exposure through food and drinking water, as currently occurs in the Netherlands, and bladder cancer risk. However, too few studies have examined this association. Therefore, further research is needed in populations exposed to higher nitrate levels to provide more information between this potential carcinogen and bladder cancer risk.

## Figures and Tables

**Table 1:** Description of mean daily intake of nitrate in food and drinking water and estimated total nitrate intake, as well as the distribution of potential confounding factors: the Netherlands Cohort Study, 1986–1995.

Exposure variable	Cases	Subcohort
Nitrate from food (mg/day)	104.5 ± 43.4	104.5 ± 44.0
Nitrate from drinking water (mg/day)	5.3 ± 6.2	4.9 ± 6.2
Total nitrate intake	109.8 ± 44.3	109.4 ± 45.2
Potential risk factors		
Age (years)	62.5 ± 4.1	61.4 ± 4.2
Alcohol intake (g/day)	15.8 ± 17.9	10.4 ± 14.4
Coffee consumption (cups/day)	5.9 ± 3.0	5.4 ± 2.7
Tea consumption (cups/day)	3.0 ± 2.5	3.5 ± 2.5
Water consumption (L/day)	2.1 ± 0.5	2.1 ± 0.5
Total vegetable consumption (g/day)	190.8 ± 79.4	193.4 ± 83.0
Total fruit consumption (g/day)	154.3 ± 122.4	175.5 ± 119.5
Vitamin C intake (mg/day)	98.7 ± 43.8	103.3 ± 43.8
Vitamin E intake (mg/day)	14.1 ± 6.3	13.4 ± 6.2
Smoking amount (cigarettes/day)^a^	17.8 ± 11.1	15.2 ± 10.2
Smoking duration (years)^a^	37.1 ± 11.6	31.7 ± 12.3
Sex (% male)^b^	766 (86.2)	2,166 (49.1)
Cigarette smoking (% ever)^b^	780 (87.7)	2,827 (64.1)
Current cigarette smoking (% yes)^b^	393 (44.2)	1,250 (28.1)
Family history of bladder cancer (% yes)^b^	10 (1.1)	85 (1.9)
High risk occupation (% yes)^b^	7 (0.8)	17 (0.4)

Values shown are mean ± SD, except where indicated.

a Among ever smokers only.

b Values shown are number (%).

**Table 2:** RRs (95% CIs) for bladder cancer in relation to quintiles of nitrate exposure in age- and sex-adjusted and multivariable analyses: the Netherlands Cohort Study, 1986–1995.

Nitrate exposure [range (median), mg/day]	Cases	Subcohort (PY)	RR (95% CI)^a^	RR (95% CI)^b^	RR (95% CI)^c^
Food					
2.0–69.0 (57.4)	168	8,512	1.00 (Reference)	1.00 (Reference)	1.00 (Reference)
69.0–88.0 (78.6)	186	8,652	1.14 (0.89–1.45)	1.16 (0.90–1.50)	1.15 (0.89–1.50)
88.0–107.5 (97.8)	181	8,706	1.00 (0.78–1.27)	1.04 (0.80–1.34)	1.05 (0.81–1.37)
107.5–135.3 (119.5)	180	8,707	1.02 (0.80–1.30)	1.06 (0.82–1.37)	1.05 (0.81–1.37)
135.3–451.1 (158.9)	174	8,564	1.01 (0.79–1.29)	1.06 (0.81–1.37)	1.04 (0.80–1.36)
*p*-Trend			0.72	0.96	0.96
Increment in 10 mg/day			1.00 (0.98–1.01)	1.00 (0.98–1.02)	1.00 (0.98–1.02)
Drinking water					
0–0.9 (0.5)	167	8,421	1.00 (Reference)	1.00 (Reference)	1.00 (Reference)
0.9–2.4 (1.4)	147	8,386	0.86 (0.66–1.10)	0.84 (0.64–1.11)	0.84 (0.64–1.11)
2.4–4.4 (3.4)	185	8,476	1.10 (0.86–1.40)	1.11 (0.86–1.44)	1.11 (0.86–1.44)
4.4–7.7 (5.6)	179	8,530	1.05 (0.82–1.34)	1.08 (0.84–1.40)	1.08 (0.84–1.40)
7.7–92.7 (10.6)	193	8,515	1.11 (0.87–1.41)	1.06 (0.82–1.37)	1.06 (0.82–1.38)
*p*-Trend			0.14	0.23	0.24
Increment in 10 mg/day			1.10 (0.98–1.23)	1.09 (0.96–1.24)	1.09 (0.96–1.24)
Total nitrate					
2.4–72.7 (60.5)	159	8,297	1.00 (Reference)	1.00 (Reference)	
72.7–92.9 (83.2)	179	8,496	1.20 (0.93–1.53)	1.26 (0.97–1.64)	
92.9–112.7 (102.4)	189	8,606	1.11 (0.87–1.42)	1.18 (0.91–1.53)	
112.7–140.8 (125.0)	174	8,530	1.08 (0.84–1.39)	1.17 (0.89–1.52)	
140.8–525.4 (165.4)	170	8,399	1.03 (0.80–1.32)	1.09 (0.84–1.42)	
*p*-Trend			0.86	0.77	
Increment in 10 mg/day			1.00 (0.98–1.02)	1.00 (0.99–1.02)	

PY, person-years.

a Adjusted for age (years) and sex.

b Adjusted for age (years), sex, current smoking (yes/no), smoking amount (cigarettes/day), and smoking duration (years).

c Adjusted for age (years), sex, current smoking (yes/no), smoking amount (cigarettes/day), smoking duration (years), and nitrate exposure from food (for drinking water analyses only) or nitrate exposure from drinking water (for food analyses only).

**Table 3:** RRs (95% CIs) for bladder cancer in relation to nitrate exposure from food, with respect to dietary vitamin C and E intake (low/high intake) and cigarette smoking (never/ever): the Netherlands Cohort Study, 1986–1995.

	Nitrate intake from food					
		Low (≤ 97.9 mg/day)		High (> 97.9 mg/day)		
	Range (mg/day)	Cases	Subcohort (PY)	Cases	Subcohort (PY)	RR (95% CI)
Vitamin C						
Low	≤ 96.5	311	14,490	167	7,063	1.00 (0.78–1.26)^a^
High	> 96.5	141	7,075	270	14,513	0.84 (0.65–1.08)^a^
Vitamin E						
Low	≤ 12.2	248	12,679	154	8,629	0.89 (0.69–1.14)^a^
High	> 12.2	204	8,886	283	12,947	1.00 (0.80–1.26)^a^
Cigarette smoking						
Never		64	7,460	45	7,336	0.70 (0.46–1.08)^b^
Ever		388	14,105	392	14,239	0.98 (0.82–1.17)^c^

PY, person-years.

a Adjusted for age (years), sex, current smoking, smoking amount (cigarettes/day), smoking duration (years), and nitrate exposure from drinking water.

b Adjusted for age (years), sex, and nitrate exposure from drinking water.

c Adjusted for age (years), sex, smoking amount (cigarettes/day), smoking duration (years), and nitrate exposure from drinking water.

**Table 4:** RRs (95% CIs) for bladder cancer in relation to nitrate exposure from drinking water, with respect to cigarette smoking (never/ever): the Netherlands Cohort Study, 1986–1995.

	Nitrate intake from drinking water				
	Low (≤ 3.4 mg/day)		High (> 3.4 mg/day)		
	Cases	Subcohort (PY)	Cases	Subcohort (PY)	RR (95% CI)
Cigarette smoking					
Never	58	7,692	45	6,764	0.99 (0.64–1.53)^a^
Ever	352	13,392	416	14,480	1.13 (0.94–1.35)^b^

PY, person-years.

a Adjusted for age (years), sex, and nitrate exposure from drinking water.

b Adjusted for age (years), sex, smoking amount (cigarettes/day), smoking duration (years), and nitrate exposure from food.

**Table 5:** RRs (95% CIs) for noninvasive and invasive transitional cell carcinoma of the urinary bladder in relation to nitrate exposure via food and drinking water and estimated total nitrate exposure: the Netherlands Cohort Study, 1986–1995.

		Noninvasive (Tis, Ta, T1)			Invasive (T2–4)		
Nitrate exposure	Range (mg/day)	Cases	Subcohort (PY)	RR (95% CI)	Cases	Subcohort (PY)	RR (95% CI)
Food							
Low	≤ 97.9	228	20,557	1.00 (Reference)	246	20,623	1.00 (Reference)
High	> 97.9	223	20,644	0.91 (0.73–1.12)	225	20,527	0.91 (0.74–1.12)^a^
Drinking water							
Low	≤ 3.4	214	20,198	1.00 (Reference)	213	20,143	1.00 (Reference)
High	> 3.4	230	20,237	1.05 (0.85–1.30)	246	20,227	1.14 (0.92–1.41)^a^
Total nitrate							
Low	≤ 102.4	220	20,157	1.00 (Reference)	234	20,187	1.00 (Reference)
High	> 102.4	224	20,278	0.97 (0.79–1.20)	225	20,183	0.96 (0.78–1.19)^b^

PY, person-years.

a Adjusted for age (years), sex, current cigarette smoking, smoking amount (cigarettes/day), smoking duration (years), and nitrate exposure from food (for drinking water analyses only) or nitrate exposure from drinking water (for food analyses only).

b Adjusted for age (years), sex, current cigarette smoking, smoking amount (cigarettes/day), and smoking duration (years).
